# An Integrative Perspective on Interpersonal Coordination in Interactive Team Sports

**DOI:** 10.3389/fpsyg.2017.01440

**Published:** 2017-08-28

**Authors:** Silvan Steiner, Anne-Claire Macquet, Roland Seiler

**Affiliations:** ^1^Institute of Sport Science, University of Bern Bern, Switzerland; ^2^Institut National du Sport, de l'Expertise et de la Performance (INSEP) Paris, France

**Keywords:** teamwork, shared mental model, affordance, group action, cognition, theory, environment

## Abstract

Interpersonal coordination is a key factor in team performance. In interactive team sports, the limited predictability of a constantly changing context makes coordination challenging. Approaches that highlight the support provided by environmental information and theories of shared mental models provide potential explanations of how interpersonal coordination can nonetheless be established. In this article, we first outline the main assumptions of these approaches and consider criticisms that have been raised with regard to each. The aim of this article is to define a theoretical perspective that integrates the coordination mechanisms of the two approaches. In doing so, we borrow from a theoretical outline of group action. According to this outline, group action based on a priori shared mental models is an example of how interpersonal coordination is established from the top down. Interpersonal coordination in reaction to the perception of affordances represents the bottom-up component of group action. Both components are inextricably involved in the coordination of interactive sports teams. We further elaborate on the theoretical outline to integrate a third, constructivist approach. Integrating this third approach helps to explain interpersonal coordination in game situations for which no shared mental models are established and game situations that remain ambiguous in terms of perceived affordances. The article describes how hierarchical, sequential, and complex dimensions of action organization are important aspects of this constructivist perspective and how mental models may be involved. A basketball example is used to illustrate how top-down, bottom-up and constructivist processes may be simultaneously involved in enabling interpersonal coordination. Finally, we present the implications for research and practice.

## Introduction

Interpersonal coordination is of primary relevance whenever sports teams perform interactively. The term “coordination” refers to the dynamic arrangement of contributing units to achieve a larger function (Gorman, 2014) and includes the organizing of team members' interdependent actions in regard to sequence and timing (McEwan and Beauchamp, 2014; see also Salas et al., 1995; Marks et al., 2001; Rousseau et al., 2006; Eccles and Tran, 2012). When teams succeed in coordinating their aggregated resources effectively, they can optimize the parameters that are relevant to their performance. One example of this is an enhanced area coverage in defensive football situations. Another example is the optimized distribution of team network nodes, which improves passing opportunities for the members of a team. In practice situations, interpersonal coordination can be established through centralized monitoring by a coach who provides external feedback in real time (Seiler, 2014). This feedback can include upcoming play selections or adjustments in location and timing (Eccles and Tran, 2012). During a competition, however, interpersonal coordination is usually not based on guidance by one central authority (e.g., the coach). Distracting noises, distance and rule restrictions can prevent teams from being directed by external feedback. In such cases, more distributed or decentralized communication channels become important (Pedersen and Cooke, 2006; LeCouteur and Feo, 2011; Passos et al., 2011; Seiler, 2014). In the competitive setting of many team sports, a high physical workload and, most importantly, time constraints impede communication-based action regulation via closed feedback loops (Cannon-Bowers and Bowers, 2006). Understanding how coordination can nonetheless be achieved in these situations is important. However, the reciprocal and dynamic relationship between the social and individual factors involved in interpersonal coordinative processes make it obvious that human interaction in social contexts is among the most complex challenges to scientific understanding (Vallacher and Nowak, 1997; see also Birrer and Seiler, 2008; Duch et al., 2010; Carron et al., 2012; McEwan and Beauchamp, 2014).

Various perspectives and empirical approaches have emerged with which to explain interpersonal coordination in team sports. Two of these approaches are central to this article. The first is the concept of shared mental models (e.g., Cannon-Bowers et al., 1993; Eccles and Tenenbaum, 2004; Cannon-Bowers and Bowers, 2006). We refer to the second approach as the ecological perspective. This perspective highlights the importance of the information sources provided by the environmental context within which a behavior is performed (e.g., Araújo et al., 2006). This article begins by outlining the general assumptions of both perspectives and their associated criticisms. It then explains why both offer indispensable information with which to understand interpersonal coordination in sports teams and illustrates the need for an integrative perspective. In our attempt to integrate the central tenets of both these perspectives into a unified view of interpersonal coordination in team sports, we borrow from a theoretical outline of group action (Cranach et al., 1986). We elaborate on this theoretical outline to integrate a third perspective. This perspective focuses on the cognitive constructive organization of the situational game context. We argue that this third perspective is necessary to explain interpersonal coordination in situations for which no shared mental models are established and task situations that remain ambiguous in terms of perceived affordances. A basketball game sequence illustrates the theoretical considerations in an applied example. The article ends with concluding remarks and the implications of the presented perspective for research and applied practice.

### Theories of shared mental models

Theories involving concepts of shared mental models are rooted in a social-cognitive framework (Eccles and Tenenbaum, 2004, 2007). They build on the key tenet that the organization of individual and team behavior involves knowledge-based mental models (Rentsch and Davenport, 2006; Araújo and Bourbousson, 2016). According to theories of shared mental models, interpersonal coordination builds on individual team members' regulating their contributions based on inter-individually shared ground. Sharedness, within this line of research, has been referred to as the synergistic aggregation of the team members' mental functioning, especially in terms of similarity and complementarity (Langan-Fox et al., 2004; see also Levine et al., 1993; Klimoski and Mohammed, 1994; Hutchins, 1995; Cohen and Bailey, 1997; Mathieu et al., 2000; Cooke et al., 2003; Reimer et al., 2006; Stanton et al., 2006; Ward and Eccles, 2006). The development of shared mental models is assumed to improve team performance by enabling nonverbal interactions and implicit coordination (Cannon-Bowers and Bowers, 2006; Rico et al., 2008; Blickensderfer et al., 2010; Cooke et al., 2013). Overt interaction between the various team members thus becomes redundant.

We focus on two factors that are believed to be involved when shared mental models facilitate interpersonal coordination. The first is the feeding forward of behavioral instructions for defined game situations (Eccles, 2010). Plans (Schank and Abelson, 1977) have often been mentioned in this connection. In team sports, macrolevel plans refer to overall team plans and strategies (Eccles and Tenenbaum, 2007). Microlevel plans include more detailed information about the individual operations required in given situations. Plays in American football are prototypical of plans at the microlevel of team operations (Eccles and Tenenbaum, 2007). While microlevel plans further specify and confine behavior, they too must be adapted to the characteristics of the situational game context (Eccles and Tenenbaum, 2007; Macquet and Kragba, 2015; Gershgoren et al., 2016). We adopt the term “top-down” to indicate that knowledge-based shared mental models feed forward information that leads to interpersonal coordination.

In a questionnaire-based study investigating shared mental models in ice hockey and handball teams, Giske et al. (2015) found support for the existence of common attack patterns specific to certain kinds of game constellations. Overall, however, empirical sports studies using shared mental models remain scarce (Gershgoren et al., 2013).

For team plans to feed forward behavioral instructions, they must exist prior to the athletes' involvement in specific game situations. Because the situational game context is dynamic and may often be unique in its configurational setting, pure reliance on pre-existing and shared plans will not always be possible (Araújo et al., 2006; Eccles and Tenenbaum, 2007; Cooke et al., 2013; Silva et al., 2013). To account for this, theories of shared mental models have posited more dynamic and implicit ways in which multiple team members' mental models can overlap in real time (Eccles and Tenenbaum, 2004; Blickensderfer et al., 2010; Eccles, 2010; Eccles and Tran, 2012). Athletes are believed to use incidentally shared knowledge of probabilities to attribute situational informers to changes in task requirements and team members' reactions to these (Ward and Williams, 2003; Williams and Ward, 2007; Eccles, 2010). The importance of multiple athletes perceiving game situations and one another's behaviors in correct, anticipative and complementary ways is highlighted (e.g., Reimer et al., 2006). Empirical support for the role of shared knowledge in the implicit coordination in team sports has been provided by Blickensderfer et al. (2010). These authors used the degree to which teammates adjust their positioning with respect to one another as an indicator of the teams' implicit coordination. They found that the degree of shared expectations of specific doubles-partner responses was correlated with teams' implicit coordination during tennis matches.

Concerns have also been raised in regard to explaining this kind of in-process coordination (Eccles and Tenenbaum, 2004) by means of shared mental models. Skepticism has been expressed concerning the reduction of team coordination processes to collective team member states (Bourbousson et al., 2011; Gorman, 2014). Sharing a common perspective on specific game situations is unlikely to occur due to differences in knowledge, skill, history and position in physical space between players (Cooke et al., 2013; see also Reimer et al., 2006; Bourbousson et al., 2011, 2012; Macquet and Stanton, 2014).

### The ecological perspective on team coordination

The approaches that we have subsumed within the ecological perspective share a focus on the environment in which team members must coordinate their behavior. In a very general sense, ecological perspectives stand in contrast with the idea of team members selecting options from those stored in mental models. Instead, ecological perspectives seek to show how the environment contributes to the kinds of interactions that occur between agents and their respective environments and to understand the properties of the environment that affect action and decision-making processes (Cutting, 1982; Greeno, 1994; Araújo et al., 2006; Araújo and Davids, 2009; Fajen et al., 2009; Vilar et al., 2012). We will adopt the term “bottom-up” to indicate that information from the environment leads to interpersonal coordination.

Gibson (1977) coined the ecological perspective by introducing the concept of affordances. By definition, affordances are opportunities to act that are directly perceivable in the environment in the here and now. By building a dynamic transactional system with their environment, athletes may perceive the environment's intrinsic meaning for behavior in terms of the environment's functional relationship to themselves (Gibson, 1979; Araújo et al., 2006). Because this is assumed not to require cognitive mediation, the role of mental models is subordinated (Gibson, 1977; see also Greeno, 1994; Araújo et al., 2006; Fajen et al., 2009).

The concept of affordances has been adopted to explain interpersonal coordination in sports teams (Silva et al., 2013). Here, the environment refers to the situational game context, which continuously changes with the behavior of the team members and their opponents. The situational game context thus constantly lays out new temporary environments and constrains the team members' possibilities in terms of coordinating their actions toward the achievement of performance goals from moment to moment. For example, previous actions will impact the options for moving on in the future. An inexact pass, a badly chosen path on the playing field or inappropriate positioning can all affect the options for action available at any given point in time (Nitsch, 2009). The remaining options that afford ways of approaching a team goal in common facilitate interpersonal coordination in a bottom-up fashion (Araújo et al., 2006). Thus, affordances are highlighted as the organizing elements that continuously provide information about how team members can coordinate within the situational game context (Fajen et al., 2009).

Empirical support for the role of the situational game context in decision making during interactive team sports has been provided by Correia et al. (2012). Using a simulated 3 vs. 3 rugby task, they found that gaps opening in particular running channels in the defensive line influenced the ball carriers' decisions to pass to either Team Member 1 or Team Member 2 or run with the ball. In a study analysing passing behavior in real-world soccer competitions, Steiner et al. (2017) found that passes were affected by the team members' positioning relative to the ball carrier, the openness of passing lanes leading to team members and the team members' degree of defensive coverage by opposing players. The findings indicate the athletes' recurring use of the same perceptual information to make passing decisions.

We should mention that the guiding role of the situational game context has also been emphasized in conjunction with perspectives that do not explicitly restrict the relationships between agents and their social context to perceptual means (e.g., Gorman, 2014; McNeese et al., 2016). According to such perspectives, the causal mechanisms of team coordination lie in the dynamic process of team interaction (Gorman, 2014). This dynamic process may include reciprocal communicative acts between team members.

If athletes perceive multiple affordances within a situational game context, this situational game context remains ambiguous, and behavior is virtually unconstrained by the perceived affordances (Cutting, 1982). Ecological perspectives have been criticized as being unclear about how specific affordances for interpersonal coordination are selected from a multitude of possibilities (Norman, 1999; Beek, 2009; Nitsch, 2009). Furthermore, the observation of two performers coordinating their behaviors with one another does not clarify what the perceived affordance was for either performer or what information constrained the link between them (Araújo and Bourbousson, 2016). To address these criticisms, it has been proposed that the notion of people as agents in ecological theories should not be reduced to the bare person-environment relationship (Cutting, 1982; Nitsch, 2009). Instead, the organizing principles of perception and situational orientation should also be applied to the processes that operate within the actors (Cutting, 1982; Greeno, 1994; Gobet, 1998; Didierjean and Marmèche, 2005; Nitsch, 2009). For example, rather than endorsing or rejecting the roles of cognition and internal representations programmatically, Nitsch (2009) has called for specifying the conditions under which they might or might not be useful. We will take up on this notion in Section Elaborating on the Outline.

### Illustrating the need for an integrative perspective

Lately, there has been a growing call for an integrative perspective on interpersonal coordination. For example, McNeese et al. (2016) state that individual and shared mental models are important because not all actions in interdependent team sports are directed by the environment. They argue for the necessity of better integrating perspectives on shared mental models and ecological perspectives to capitalize on the strengths of each in the understanding of interpersonal coordination in sports teams. Gorman (2014) states that a general theory of interpersonal coordination should involve intention and knowledge on the part of team members while also considering environmental constraints as fundamental to interpersonal coordination (see also Araújo et al., 2006; Pedersen and Cooke, 2006; Duarte et al., 2012; Cooke et al., 2013).

To illustrate the need for an integrative perspective in the context of team sports, one can recall how often strategies and plans are discussed in practice sessions (Gershgoren et al., 2013; Giske et al., 2015). Teams practice defensive behaviors, specific strategic alignments in response to the opposing team's behavior, to near perfection. Offensive plays to be announced during games are also rehearsed. This kind of pre-process coordination (Eccles and Tenenbaum, 2004, 2007), which builds up shared mental models, is so omnipresent in team sports that excluding it from a theory of interpersonal coordination will certainly result in painting an incomplete picture. Furthermore, it is common practice to rehearse specific modules of coordinated team plays, which can be flexibly adapted to many game situations. So-called “give-and-goes” are an example from basketball. In a give-and-go, an athlete passes (gives) the ball to a team member. The athlete then immediately runs (goes) to a new spot to offer himself as an opportunity to pass the ball again (Eccles and Groth, 2007). In soccer, players practice dynamically positioning themselves in a triangular alignment. This way, passing opportunities for the ball carrier can constantly be maintained (Giske et al., 2015; for further examples, see Eccles and Tran Turner, 2014).

On the other hand, a theory of interpersonal coordination must incorporate the fact that coordination always occurs within specific and sometimes unpredictable game contexts. Hence, the role of the information provided by that situational game context is paramount. Sometimes, athletes who are perceptually attuned to their team and game contexts may be able to perceive these contexts directly by means of the acts they afford. For example, Fajen et al. (2009) point out how an open passing lane to a team member affords a pass to this team member. At the same time, passing lanes that are well-defended by opposing players perceivably constrain passes (Steiner et al., 2017).

In this article, interpersonal coordination that is directed by shared mental models or enabled by the perception of affordances frames our integrative perspective. We argue that team coordination is also established in situations for which shared mental models are not established and situations in which athletes cognitively process the information provided by the situational game context. Indications that such situations do occur in the context of team sports can be seen in a line of qualitative research involving interview techniques such as video-stimulated recall (e.g., Sève et al., 2005; Poizat et al., 2009; Bourbousson et al., 2010, 2011, 2012). Video recordings of team behavior in natural settings and verbalizations during post-match interviews are used to understand how individuals construct meaning in game situations. These retrospective verbalizations indicate that in mental models, sharedness is not always achieved (e.g., Poizat et al., 2009; Bourbousson et al., 2011, 2012). They further indicate that athletes take into account multiple situational factors, mobilize prior knowledge and combine these to construct new knowledge about the situational game context (Sève et al., 2005; Poizat et al., 2009; Bourbousson et al., 2011).

In the following sections, we will develop an integrative perspective on interpersonal coordination in interactive team sports. This integrative perspective has no intention of altering or criticizing existing theories. Instead, team coordination *exclusively* directed by shared mental models, as opposed to that *exclusively* directed by the perception of affordances, will serve as the theoretical poles of the integrative work. In Section A Theoretical Outline of Group Action, we summarize the theoretical outline of group action by Cranach et al. (1986), which serves as a framework for our integrative perspective. The outline views group action as both directed by team plans and reactive to the situational game context. Thus, both top-down and bottom-up processes play important roles in the regulation of team behavior. In Section Elaborating on the Outline, we further elaborate on Cranach et al.'s outline (1986) and explain how interpersonal coordination can be established in situations that do not fit either of the theoretical poles. Following Nitsch's (2009) call, we have considered the ways in which information from the situational game context can contribute to the emergence of interpersonally coordinated behavior in ways that go beyond the information's most direct link to agents via the perception system. The roles of mental models in the subjective organization of situational opportunities to co-act will therefore be discussed.

## A theoretical outline of group action

Cranach et al. (1986) consider teams to be self-active systems that actively direct their behavior toward certain ends. The impact of external factors (e.g., through the perception of situation-specific information) is considered an integral part of directed behavior. However, team action is not affected only by external information. It is also instantaneously guided by internally stored information (e.g., cognitively represented team plans). Thus, perceptual and cognitive processes are both involved in the system's monitoring of external contexts and the steering of behavior.

Cranach et al. (1986) argue that because it is based on individual goal-directed behavior, team action possesses the same characteristics as individual action but is complemented by additional features that stem from its social nature. These additional features are communicative and cooperative processes that become possible and necessary through the involvement of multiple persons. Cranach et al.'s theoretical outline of group action is built on four central components: the *structure of the task*, the *structure of the team*, the *information-processing structure* and the *action execution*.

The structure of the task and the structure of the team are essential in defining the conditional framework for team efficiency. For optimal performance, team and task structures must be in accord with one another. Cranach et al. (1986) define a *task* as a social demand that requires an actor to act. For the most part, tasks are closely related to specific ecological settings (e.g., a specific game situation). Insofar as the task contains detailed information about goals and plans, Cranach et al. speak of a task *structure*. By this definition, task structures are not determined simply by the information available in specific game situations. Instead, the information available in the situational game context is complemented with internalized scenarios, e.g., mental models that include goals and goal-directed plans that are viable means of task performance.

Team structures, on the other hand, are associated with the formation of a team, including the assignment of all team members to specific task-relevant functions and the relationships between team members during their involvement in a single interactive task. In some sports (e.g., sailing), the team structure is clear because the team members' roles are distinctly attributable to a set of predefined subtasks. In most interactive sports, however, general role assignments do not predefine specific functions in all situations down to the last detail. Instead, the required functions must be specified in relation to the constraints of situational game contexts, which often appear at short notice. Thus, team members must adapt their behavior according to the current task structures.

The *information processing structure*, the third component of the model, describes the processes underlying the team members' adaptation to changing task structures. Team-action-related information processing takes place at both the individual and team levels. On the level of individual team members, the theory considers cognitive information processing, which is viewed as a unique instrument for the mental guidance of goal-directed action.

Communicative processes complement cognitive information processing in individuals. Cranach et al. (1986) refer to this as information processing at the team level. Commands and assisting calls represent the flow of information between team members^1^. Moreover, the communication of an individual perception can affect the situational orientation of the team^2^. Cranach et al. also note that communication enables teams to learn action schemata for future acts. This exactly corresponds to the kind of pre-process coordination referred to by Eccles and Tenenbaum (2004, 2007).

The model's fourth component is *action execution*. Team behavior consists of individual acts and social execution. Appropriately executed and mutually coordinated individual acts allow interpersonal coordination to emerge at the team level. Cranach et al. (1986) argue that individual action is organized along three dimensions: hierarchy, sequence and complexity. While the authors explain the dimensions' relevance to individual acts, we will later illustrate how the same dimensions can be considered as organizing dimensions of interpersonally coordinated team behavior.

Finally, Cranach et al. (1986) argue that groups often perform within equifinal task situations (Heider, 1958). Equifinality refers to the fact that the completion of complex team tasks is usually not restricted to one unique solution. Instead, many potential paths conceivably allow attaining the same goal (see also Oesterreich, 1981). This flexibility can be advantageous because it enables teams to approach given requirements in light of existing team abilities. On the downside, equifinality complicates the emergence of interpersonal coordination because it increases the degrees of freedom. Furthermore, the behavior of opposing teams becomes more unpredictable.

## Elaborating on the outline

To elaborate on our view of the involvement of internal information in the subjective construction and organization of the information provided by the situational game context, we adapt and extend Cranach et al.'s (1986) ideas. We will discuss two factors we consider central in regard to this internal information: organizational rules and the contents of mental models.

### Organizational rules

In order to explain how situational opportunities for interpersonally coordinated team behavior are established through a team member's interaction with a situational game context, we adopt Cranach et al.'s (1986) notion of the three-dimensional organization of action and apply it to the organization of interpersonally coordinated team action. Because it is directed toward the attainment of primary team goals, individual behavior requires reactive adjustment to the situational game context and constant (re-)organization along hierarchical, sequential and complex dimensions. While all three dimensions of action organization supposedly act in combination, we will briefly explain their features separately.

The hierarchical aspect of team action organization refers to monitoring a situation's functional relationship to the attainment of the primary goals that are currently directing behavior. From an athlete's point of view, this includes determining a situation's offerings in regard to the highest task goals, which provide the athlete's directional perspective (Araújo et al., 2006; see also Klein et al., 2007; Nitsch, 2009). In team sports, this directional perspective is set out by the general rules of the specific sport. If the main objective is to score more points than the opposing team, then scoring points and preventing opponents from doing so define the two goals that direct behavior at the highest level of the hierarchy. The team must adopt a structure (e.g., assign functions to team members) and perform behaviors that are optimally suited to attaining these goals.

During task performance, the situational constraints organized by the opposing team may block instant paths to the primary goal of scoring. Consequently, situational game contexts must be monitored concerning the sequentially and complexly organized goal approximations they allow. Preparatory steps, such as bringing the ball to a shooting position nearer to the target, become necessary (Oesterreich, 1981). These preparatory steps give rise to subgoals at lower levels of the hierarchy. Subgoals are abandoned when achieved and then replaced by those that follow in the sequential alignment toward higher-order goals (Wilensky, 1983). If the situational game context changes in a way that enables a more direct path to higher-order goals, then temporary subgoals may be abandoned before they have been reached (Oesterreich, 1981). The relationship between lower- and higher-level goals determines the hierarchical-sequential organization of team behavior (Volpert, 1982; Cranach et al., 1986; Marks et al., 2001; see also Hacker, 2005).

The dimension of complexity complements the goal-directed organization of team behavior. We extend Cranach et al.'s (1985, 1986) use of the term (they use it to describe multiple simultaneously performed acts of a single individual, e.g., moving one's head and feet) and use it to refer to the simultaneous behavior of *multiple* team members (see also Marks et al., 2001). The dimension of complexity in organizing team action refers to perceiving or creating opportunities to co-act with others. Simply put, it is relevant in relation to the question, “Can I attain a current action goal by myself?” When the answer is no, this dimension of action organization becomes relevant. Let us assume a team is in possession of the ball and striving to position itself to attempt to shoot. The ball carrier brings the ball down the wing. He monitors the situation for potential pass receivers because he plans to play a cross to bring the ball closer to the goal. At the same time, some players are running to position themselves in the box. They have seen the ball carrier and anticipate an opportunity to complement his efforts. This must occur simultaneously with the person who has the ball looking for a position from which he can play a cross-court pass. Another example is a through ball, in which case the intended receiver of a pass should be underway by the time the ball is kicked to the future point of reception.

We argue that the monitoring of the situational game context in regard to these three dimensions is part of a subjective, constructive process that enables interpersonally coordinated team behavior in situations with no shared mental models and no or no unambiguous affordances.

### Mental representations in the specification of situational context

When monitoring the situational game context, athletes may need to rely on mental models (internal information). Numerous examples of mental models assumed to be relevant in the domain of team sports have been provided: plans and patterns of coordination (Eccles and Tenenbaum, 2004, 2007; Macquet, 2009; Macquet and Kragba, 2015), specific behavioral programs, scripts (Schank and Abelson, 1977), and mental models about other team members (Annett, 1996; Rentsch and Davenport, 2006; Gershgoren et al., 2013), to name only a few (Rouse et al., 1992; Reimer et al., 2006; see also Eccles and Groth, 2007).

To illustrate the contribution of mental models to the monitoring of the situational game context and the subjective construction of opportunities to act, we will consider mental models about other team members. Such mental models have been defined as consisting of, among other things, knowledge about the specific strengths and weaknesses of other team members (Annett, 1996; Reimer et al., 2006; Rentsch and Davenport, 2006; Gershgoren et al., 2013). In team sports, team members are part of the situational game context and represent perceivable external information. An athlete's mental models, MEMBER A and MEMBER B, of her Team Members A and B enable the inclusion of additional information in the situational game context. The mental models themselves need not include other situational factors. While such mental models are restricted in terms of content, they are useful in various game contexts because they are flexibly transferable to various situations and can be combined with other mental models (Macquet, 2009; Macquet and Kragba, 2015). If the mental model MEMBER A includes information about the excellent technical skills of Team Member A, then Team Member A will be considered a potential pass receiver in numerous game situations (Johnson, 2006). Team Member A's status as a potential passing opportunity is thus characterized by a certain level of stability. However, this status is not a permanent attribute of Team Member A. Instead, it also depends on other situational features. If Team Member A is being defended well, the extent to which she represents a passing opportunity requires reappraisal, considering both her abilities (included in MEMBER A) and her current defensive coverage (information provided by the situational game context). In combination, these various sources of information determine a team member's current state as a passing opportunity. According to representational theories of the mind, team members can combine a large but finite number of mental models in numerous ways to create increasingly complex mental models (Margolis and Laurence, 2007). Similarly, we propose that mental models can be combined with external information sources in numerous ways to specify the constraints and opportunities within increasingly complex game contexts. Thus, opportunities to act may be detectable because of the highly elaborate mental model that agents hold of the current situational game context (e.g., a specified team plan to be followed in the given situation). However, athletes may also detect opportunities to act when localizing specific information sources that appear as subcomponents of a complex situational game context (e.g., a team member standing open).

Referring to the great importance Cranach et al. (1986) placed on the above-mentioned structures in understanding interpersonally coordinated team behavior, Seiler (2014) categorizes mental models according to their relatedness to task structure(s), team structure(s), intrateam communication and cooperation. In our example, we will adopt this proposal. We want to stress that the content assigned to one of these four structures is not always clear-cut and that the categories are by no means terminal.

Mental models that relate to task structure can include the macro-environment of a specific sport. This term refers to the general framework provided by the rules of the sport (Kaminski, 2009). Athletes must constrain their actions to this specific framework. Mental models that are linked to individual and role-specific tasks have much in common with the concept of taskwork knowledge (McIntyre and Salas, 1995).

Mental models that relate to group structure may include the positioning and strategic alignment of team members, formal role assignments, roles that are established by means of team members' task-relevant strengths and weaknesses and informal roles (e.g., Annett, 1996; Eccles and Tenenbaum, 2004; Rentsch and Davenport, 2006; Gershgoren et al., 2013).

Mental models related to team communication may refer to communicative signs that announce specific plays. They may also include hand signals indicating a player's availability to receive a pass. Teams often use dedicated signs to inform one another about planned moves (Eccles, 2010; Macquet and Kragba, 2015). Because communication in interactive team sports is often visible to all, attempts to conceal information from the opponent include special communication systems and signs. Team members with a common history in sports may have developed mental models of one another's behavioral idiosyncrasies. This helps players to “read” their team members by using nonverbal channels of communication (e.g., Eccles and Tenenbaum, 2004).

Mental models related to interpersonal coordination have much in common with the concept of teamwork knowledge (McIntyre and Salas, 1995; Bowers et al., 1997; Eccles and Tenenbaum, 2004). These models include information about how the actions of multiple individuals can be successfully integrated to produce group-level performance. Overall team tactics, game plans and scripts for specific situational game contexts are examples of this (Rentsch and Davenport, 2006). These models can also refer to plans with various levels of detail (Eccles and Tenenbaum, 2007). They may be taught explicitly (e.g., through coaching) or developed based on experience in real game situations (Annett, 1996; Eccles, 2010).

## An example from basketball

**Figure 1:**
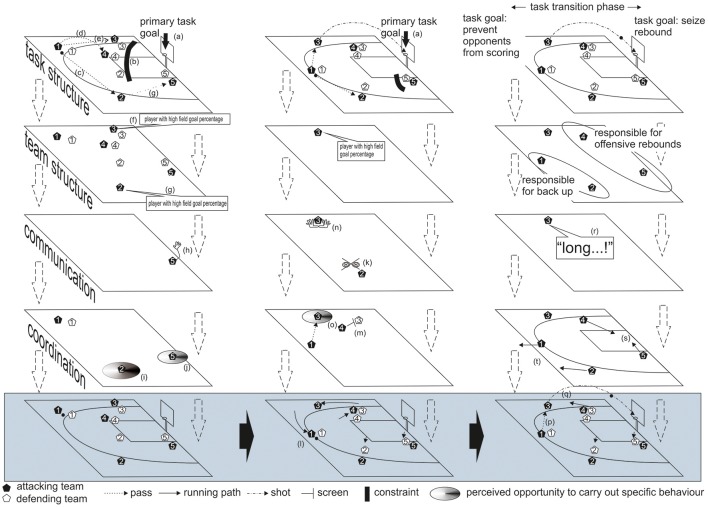
Three-frame sequence of an evolving basketball game situation. Annotations in the text.

Figure 1 exemplifies the involvement of mental models in the subjective specification of the situational game context. The scenes present an offensive situation in basketball. They include three chronologically ordered frames that illustrate the evolving game. The three frames highlighted in gray illustrate the game situation as it objectively presents itself to the observer. The figure further includes four levels that indicate the mental models involved in the constructive organization of the situational game context. These levels refer to mental models of the task structure, team structure, communication and coordination. The example illustrates how such models could be involved in specifying information from the situational game context and organizing the situation to reveal opportunities for interpersonally coordinated team behavior. It further shows how situation-related mental models (internal information) and perceptual or communicational (external) information can be integrated according to the hierarchical, sequential and complex dimensions of action organization.

In the initial frame, all athletes have taken their positions, as defined by the team's formation (this refers to the feed-forward function of shared mental models). The offensive team is shown in black, and the defense is shown in white. In basketball, the primary goal of the team in possession of the ball is to score baskets (a). All team members share this group goal and use it to direct their behavior on a global level (e.g., Reimer et al., 2006; Wieber et al., 2012). The point guard (Black #1) is in possession of the ball. Situational constraints prevent him from attaining the primary task goal directly. Often, these constraints are created by opponents who attempt to neutralize the goal-directed efforts of their adversaries. In the current example, the tight defense created by the guard's direct opponent (White #1) does not allow a promising attempt at a long-distance shot. Moreover, three other opponents (White #2, #3, #4) are ready to back up Defender 1 if the point guard attempts to get past him and penetrate into the zone (b). Preparatory steps are required to approximate the hierarchically higher goal sequentially. The team's behavior is directed toward scoring a basket, but in this case, it is adapted to the situational constraints and (re-)organized in a hierarchic and sequential order.

Guided by the newly adopted approximation goal of preparing a shooting possibility for the team, the situation offers the guard three options in terms of passing the ball to a team member (c, d and e). All three options could potentially lead to scoring a basket. This indicates the task situation's equifinality. In choosing one option, mental models about the other team players come into play (f and g). The point guard knows that both the shooting guard (Black #2) and the small forward (Black #3) have high field goal percentage from behind the three-point line. Based on his knowledge about his team members, no option emerges as being superior to the other. Passing the ball to either of them will enable an equally good goal approximation. The shooting guard's defender (White #2) is located at the high post. This leaves enough room for a direct pass to the shooting guard. The shooting guard's skills, in combination with the loose defense of his opponent, become integrated into a contextual opportunity to pass him the ball. Simultaneously, the center (Black #5) takes his position and calls for the ball by raising his arm (h). Passing the ball to the center directly is not an option, because of the long distance involved, combined with a bad passing angle. Instead, delivering the ball to the center via the shooting guard is more favorable. In the current situation, the right side of the playing field offers the point guard better options to act in a goal-directed manner (i and j).

In the second frame, the point guard directs his behavior toward moving the ball to the right side. The shooting guard does not reciprocate the point guard's eye contact (k). This perceptual information warns the point guard that a pass might reach the shooting guard unexpectedly and thus be risky (e.g., Macquet and Kragba, 2015). Hence, the point guard starts to dribble the ball toward the shooting guard (k). The point guard's defender (White #1) follows closely and still does not allow the shooting guard to shoot the ball or penetrate into the zone. As in the first frame, the search for indirect paths to the primary goal remains necessary. The actions and reactions of the various players cause local changes in the task constraints. As the guard moves toward the right side of the playing field, the defenders of the shooting guard and the center (White #2 and #5, respectively) decrease their distances from their direct opponents. They do so to make passes from the point guard to their directly opposing players more difficult. As a consequence, the previous opportunities to pass (i and j) become less likely to lead to achieving the task goal. Meanwhile, the power forward (Black #4) has set a screen (m) for the athlete defending the small forward (White #3). This screen is not part of a predefined play calling for specific action by the whole team. However, it is a behavior module that can be flexibly adapted to many game situations. Once initiated, those who perceive it understand the steps of the behavior module. The small forward (Black #3) has recognized the opportunity created and taken it to put some distance between himself and his defender. He runs away from the zone to take a position behind the three-point line. He signals his readiness to receive the ball by putting out his hands toward the point guard (n). The point guard perceives the open team member as a passing affordance (o).

In the third frame, the point guard plays the corresponding pass (p). Immediately after receiving the ball, the small forward (Black #3) takes a jump shot (q). As the ball leaves the small forward's hands, a task transition begins. For a short time, the team task is undefined at the level of the primary task goal. If the ball falls into the basket, the primary task goal changes from scoring a basket to preventing the opponents from scoring a basket. If, however, the attempt is not successful, then there may be a chance to regain possession of the ball via offensive rebounding. By calling out that his shot will be off-target (r), the small forward informs his teammates that there will be an opportunity for an offensive rebound. The team's predetermined, shared strategy mandates that both the power forward (Black #4) and the center (Black #5) go for the offensive rebound (s), while the guard and the small forward will run back up the floor to prevent a fast break (t).

This three-frame sequence depicts a short excerpt of a basketball game. It illustrates how mental models shared by the entire team feed forward into behavioral guidelines; this refers to a top-down process. At the same time, it illustrates how information from the situational game context is used to detect opportunities to carry out specific behaviors and thus help to regulate and coordinate team behavior in the process. The pass from the point guard to the open small forward (o) was triggered by the perception of a passing affordance; this is a bottom-up process. The sequence further illustrates how mental models (internal information), in interaction with (external) information sources, enable the assignment of subjective meanings to the situational game context in a modular and constructive manner.

The involvement of mental models in specifying subjective opportunities to (inter)act has primarily been illustrated from the perspective of the point guard (Black #1) and the small forward (Black #3). We suppose that the same process of subjectively specifying a situational game context takes place for all team members. According to this view, interpersonal coordination is enabled when multiple subjective perspectives on the situational game context are constructed congruently, complementarily or reactively (e.g., Eccles and Tenenbaum, 2004; Rentsch and Davenport, 2006; Bourbousson et al., 2011). An example of congruency is the common adoption of the same goals or subgoals, which provides overall direction for personal behavior. An example of reactive complementarity can be seen in the small forward taking advantage of the screen the power forward has set. He reacts to the overt behavior of his team member, which signals the initiation of a specific module of a coordinated team play (give-and-go), and adapts his own behavior to it (Macquet and Kragba, 2015).

In the chosen example, the point guard and small forward do not share the same plans to guide their behavior from Frame 1 through Frame 3. While the entire team shares the same primary goal, the pass from the point guard to the small forward is an example of team coordination being established locally, without the entire team sharing a mental model of the current game situation (see Bourbousson et al., 2012). The situation is characteristically different after the shooting attempt. Now, all team members adapt their behavior to a shared mental plan because everyone assumes their roles as defined by the game strategy for this kind of situation. This example illustrates interpersonal coordination as it is based on the goal-directed adaptation of multiple individuals to situational game contexts. It exemplifies how top-down, bottom-up, and constructivist regulation mechanisms are all involved in this process.

## Concluding remarks

Interpersonal coordination in interactive sports is a complex phenomenon. Several streams of research approach it from different perspectives. The present article builds on the important contributions some of these approaches have made and aims to integrate and position them within a theoretical framework. This framework borrows from the theoretical outline of group action proposed by Cranach et al. (1986). According to this outline, group behavior regulated by shared team plans is an example of team behavior being directed from the top down via a team-level construct. Team behavior that emerges from athletes perceiving situational affordances is an example of how group behavior is reactive to the situational game context and is thus regulated from the bottom up. We extend the framework by integrating a perspective on the subjective construction of the situational game context. This constructivist perspective accounts for interpersonal coordination as it may be established in novel situations, for which teams do not share mental models. It further accounts for interpersonal coordination in situations with multiple perceived affordances or situations to which athletes are not sufficiently attuned in order to act based on perceived affordances. We argue that under such circumstances, opportunities for interpersonally coordinated team behavior are constructed based on the hierarchical, sequential and complex dimensions of group action organization. We further illustrate how mental models may be involved in this constructive process. For illustrative purposes, we have categorized mental models according to the four structures presented by Cranach et al. (1986). This categorization's primary purpose is to delineate the dynamic integration of multiple mental models as they connect with a given game situation. We want to stress that the categories are exemplary. Finally, an example illustrates how top-down, bottom-up and constructivist processes may simultaneously enable interpersonal coordination.

The integrative perspective's primary implication for research is that a search for *the* one regulation mechanism in the coordination of sports teams is not productive. It argues for following various approaches to better understand the coordination of interpersonal behavior. Provided that multiple mechanisms are involved in enabling interpersonal coordination, one general implication for research is the need to understand in what situations and to what degree they are involved. Hence, designs that estimate the mechanisms' relative contributions to interpersonal coordination in various game contexts are needed.

In our basketball example, the point guard's consideration to pass the ball to the shooting guard is based on both information provided by the situational game context (the shooting guard standing open) and his mental model of the shooting guard's shooting skills. When studying basketballers' real-world behavior, every team member without the ball (all representing potential passing opportunities) can be described by his relative position on the playing field. Perceptual information as available from the subjective perspective of the ball carrier can be quantified (e.g., Steiner and Kunz, 2017). Furthermore, each team member can be assigned values that indicate aspects of the ball carrier's mental models about these particular team members. For example, a high value could indicate that the ball carrier's mental model of this team members is one of a highly skilled shooter. Finally, each team member can be described in terms of the passing priority he is given by a team's playing strategy for this kind of situation. Coding passes dichotomously (the player receiving the balls is coded “1,” and all disregarded team members are coded “0”), the effects of the variables representing the information provided by the situational game context, mental models about other team members and shared team strategies can be estimated using logistic regression analyses (e.g., Steiner and Kunz, 2017).

Similar tests could be conducted using experimental designs. In virtual reality settings, the space available to the shooting guard can be manipulated (e.g., Correia et al., 2012). The skill-level of the player in the shooting guard position can be manipulated by showing team members with different levels of shooting ability. Finally, this manipulation can be performed in situations for which teams do and do not have predefined team strategies. Using three gradations for both available space and shooting skills, the experimental manipulation results in a 3 × 3 × 2 design to test the relative effects of available space (information provided by the situational game context), shooting skills (mental models about other team members) and shared team strategies.

Based on our integrative perspective, we hypothesize that passing decisions are affected by the spatial properties of the situational game context, athletes' mental models of team members and shared team plans. Testing each effect in isolation, we thus expect to find higher probabilities for passes to spatially less constrained team members, higher probabilities for passes to team members who are mentally represented as having better shooting skills, and higher probabilities for passes that are in accord with pre-defined team plans. When testing the effects simultaneously, we would, for example, expect larger effects on the part of spatial constraints on passing decisions when no team plans are available than when team plans are available. Furthermore, we expect that the effect of prioritizing passes to more-skilled team members will decrease as passes to more-skilled members become spatially constrained.

With regard to practical applications, the integrative perspective implies that there are multiple paths to interpersonal coordination. According to the provided perspective, shared team plans and strategies are an important pillar of interpersonal coordination. Coaches should enable this kind of pre-process coordination. The fact that it will not be possible to pre-define shared team plans for every kind of situation encountered does not lower their importance in all those situations for which they can be established. A second implication is that coaches should tell their athletes that there will be situations for which no pre-defined team plans are available. Preparing athletes for this kind of unpreparedness could include instructing them to look for the specific opportunities available in given situations (rather than losing time attempting to remember a non-existent plan). A third implication is the need to clearly communicate team goals. According to the organizing rules discussed, primary goals help athletes integrate the available information sources to make sense of the situational game context. Clarifying team goals enables a common denominator in this subjective organization of situational game contexts. Whether the team goal is to play aggressively or to play safely makes a difference in regard to the athletes' perspective on the game. Finally, the framework posits that athletes profit from the information they are given prior to the game. The information could include the specific strengths, prioritized running paths or defensive weaknesses of opponents. This information enables mental models athletes can associate with external information in real time to actively construct their perspective on the situational game context.

To conclude, we have examined the integrative perspective in relation to recent investigations on briefing and debriefing in elite sports. According to Macquet et al. (submitted), head coaches prepare their players by transmitting the game plan to them and providing them with information about their opponents (i.e., strengths, weaknesses, behavioral tendencies, and specific opponents to survey). This information enables team members to share team plans and establish knowledge that can be used to flexibly construct a subjective perspective on the situational game context during the course of the game. Furthermore, coaches teach team-sport players what to look for (Macquet et al., 2015). They guide the players' perceptions and enable them to perceive meaningful information. This guidance helps players read the game and better coordinate with their teammates and opponents. In our view, these findings support the presented integrative perspective. They indicate that the integrative perspective may represent a greater challenge to empirical science than to applied work. Research attempts to describe and explain the principles underlying interpersonal coordination via scientific means. This includes various approaches that may not always enable a common perspective on the phenomenon in question. In our integrative perspective, these various approaches do not strive for exclusiveness or general superiority. Combined, they contribute to a better understanding of a common focus: interpersonal coordination within interactive team sports.

## Author contributions

SS, AM, and RS drafted the work and provided approval for the version to be published.

### Conflict of interest statement

The authors declare that the research was conducted in the absence of any commercial or financial relationships that could be construed as a potential conflict of interest.

## Footnotes

^1^Note the similarity to Cooke et al's (2013) conceptualization of interactive team cognition.

^2^Note the similarity to the concept of shared situation awareness (e.g., Macquet, 2016).
